# The unsung heroes: A scoping review of the experiences of lung transplant informal caregivers

**DOI:** 10.1016/j.jhlto.2025.100472

**Published:** 2025-12-23

**Authors:** Jane Simanovski, Jody Ralph, Jamie Crawley, Kelly Bryce, Dana Sleiman, Edward Cruz

**Affiliations:** a Faculty of Nursing, University of Windsor, 401 Sunset Avenue, Windsor, Ontario N9B 3P4 Canada; b Transplant Institute, Henry Ford Health, 2799 West Grand Blvd, Detroit, MI 48202

**Keywords:** Lung transplantation, Lung transplant recipients, Informal caregiving, Caregiver experience, Scoping review

## Abstract

Informal caregivers support patients after lung transplantation (LTx). With growing recognition of the multiple demands placed on caregivers, this scoping review aimed to systematically map the literature surrounding informal caregiving experiences after LTx using JBI guidelines. Multiple databases were searched from January 2010 to May 2025 based on a combination of synonyms and controlled vocabulary related to “caregiver” and “lung transplant recipients”. A total of 404 records were screened after the removal of duplicates. Among these, 16 sources met the inclusion criteria with 12 (75%) classified as full publications, 3 (19%) peer-reviewed conference abstracts, and 1 poster presentation. Most studies were based in North America (11/16 [69%]) with the remainder from Europe or Australia. Only 19% (3/16) of the sources were published within the past 5 years. There were 7 qualitative studies (44%), 6 quantitative (38%), 2 mixed methods (12%), and 1 literature review. Informal caregivers described a wide variety of challenges ranging from high levels of caregiver burden, psychological and emotional impacts, handling multiple daily practicalities, knowledge deficits, and the need for more support. Positive experiences of the informal caregiver role include positive adjustment, presence of support networks and relationships, improved quality of life, and benefiting from educational support and preparation. Informal caregivers remain an integral resource in supporting patients after LTx. However, most available evidence predates recent advances in transplantation practice—with 80% published before 2020—limiting its current relevance and highlighting the need for further research and targeted interventions to support this population.

## Background

Lung transplantation (LTx) improves both survival and quality of life as more than 4600 LTxs are performed annually worldwide.^1^ After LTx, patients face a plethora of complications including allograft rejection, infections, and the development of additional comorbidities.^2^, ^3^ Hence, median survival for adults undergoing LTx has improved over time, now reaching 6.7 years—an increase from 4.7 years in the 1990s and 6.5 years in the 2000s.^2^ Among surviving recipients, functional outcomes vary, with only 14% returning to work in the first-year post-transplant.^4^

Given scarcity of available donors, candidate evaluation identifies risk factors for poor post-transplant outcomes—such as comorbidities, limited rehabilitation potential, and inadequate social support or caregiving plans—which may serve as an absolute or relative exclusion criteria.^5^ This support is typically provided by informal caregivers, usually spouses or partners; but may include parents, children, extended family, neighbors, or friends. Informal caregivers are individuals who offer unpaid, ongoing assistance with activities of daily living to other individuals affected by chronic illness.^6^ Chronic caregiving demands may negatively impact caregivers’ health and self-care behaviors.^7^ In a landmark prospective population-based cohort study examining demands of caregiving, participants affected by caregiving strain had 63% higher mortality risk as compared to non-caregiving controls.^7^ Caregivers of persons living with mental illness in the community found that caregivers faced financial strain, often reducing work hours or retiring early to provide care.^8^

Caregiving demands after LTx may include handling myriads of new time-sensitive medications; direct supervision; performing medical interventions (e.g., chest physiotherapy and home spirometry); communicating with transplant team; providing emotional support; and transporting to medical appointments, tests, and pulmonary rehabilitation programs. Many caregivers are forced into taking family leaves of absence, placing additional financial demands on an already stressed household.

Given a scarcity of literature surrounding caregiver experiences after LTx, this scoping review aimed to answer the following research questions:

1. What is the range of research that examined informal caregiving after LTx?
2. What are the positive and challenging experiences of informal caregivers of LTx recipients?

## Methods

### Protocol

The protocol, not registered, was developed a priori following guidelines for conducting scoping reviews by Arksey and O’Malley^9^ and refined by the Joanna Briggs Institute (JBI).^10^ JBI manual for evidence synthesis was used for step-by-step methodology^10^ and the Preferred Reporting Items for Systematic Reviews and Meta-Analyses (PRISMA)^11^ were used to provide reporting guidance. “Source” refers to the included literature in the scoping review.

### Inclusion criteria

#### Participants

This review included studies that explored experiences of informal caregivers aged 18 years or older who provide care to lung transplant recipients, regardless of ethnicity, transplant center, or presence of comorbidities. Caregivers caring for patients who had not undergone transplantation, or those who were minors were excluded.

#### Concept

For this review, the concept refers to the experiences such as perceptions, roles, and challenges encountered by informal caregivers—individuals who provide unpaid support to lung transplant recipients. They include emotional, physical, and practical aspects of caregiving and the impact of caregiving responsibilities. The concept excludes professional or paid caregivers.

#### Context and search strategy

This review included sources that reported on the experiences of informal caregivers of lung transplant recipients in any setting, such as home, community, and outpatient environments. Studies were considered regardless of geographic location, healthcare system, or type of transplant center. Evidence sources that examined caregiving for patients awaiting transplantation or for other organ transplant types without specific reference to lung transplantation were also excluded.

A health sciences librarian developed search strategies and searched multiple databases (Ovid MEDLINE, Embase, Web of Science Core Collection, CINAHL, and Ovid PsychInfo) on January 31, 2024, and updated on May 8, 2025. These databases were selected to ensure comprehensive coverage of biomedical, nursing, psychosocial, and interdisciplinary research relevant to lung transplantation and informal caregiving. Searches were based on combinations of keyword terms and controlled vocabulary related to “caregivers” and “lung transplant recipients” and limited to the English language. Preliminary searches revealed minimal publications between 2020 and 2023, due to research priorities shifting toward COVID-19 and disruptions in transplant programs. To ensure a comprehensive review, the timeframe was expanded to include studies published from 2010 onward, capturing caregiver experiences during periods of constant lung transplant trends. Studies published before 2010 were excluded as they may not reflect current caregiving realities given advances in lung transplantation and improved survival. A second health sciences librarian peer-reviewed the search strategies. An example of the Ovid MEDLINE search strategy is presented in Table 1, in accordance with PRISMA reporting guidelines.

**Table 1:** Ovid MEDLINE Search Strategy May 8, 2025

#	Query	Results from 8 May 2025
1	Caregivers/	57,528
2	(caregiv* or care giver* or carer* or care taker*).mp.	147,716
3	1 or 2	147,716
4	exp Family/ or Friends/ or adult children/ or grandparents/ or only child/ or parents/ or fathers/ or mothers/ or siblings/ or spouses/	395,655
5	(relatives or next of kin or spouse* or parent).mp.	310,051
6	4 or 5	604,360
7	3 or 6	714,511
8	exp Lung Transplantation/	19,984
9	(lung transplant* or Heart-Lung Transplant* or Liver-lung transplant* or ((cardiothoracic or thoracic) adj2 transplant*)).mp.	27,904
10	transplant recipients/ or ((transplant* or re-transplant*) adj5 (recipient* or patient*)).mp.	188,410
11	Lung/ or lung.mp.	1036,579
12	10 and 11	17,216
13	8 or 9 or 12	32,472
14	7 and 13	270
15	limit 14 to english language	254
16	limit 15 to dt=20240130-20250507	18

### Selecting the evidence

**Figure 1:**
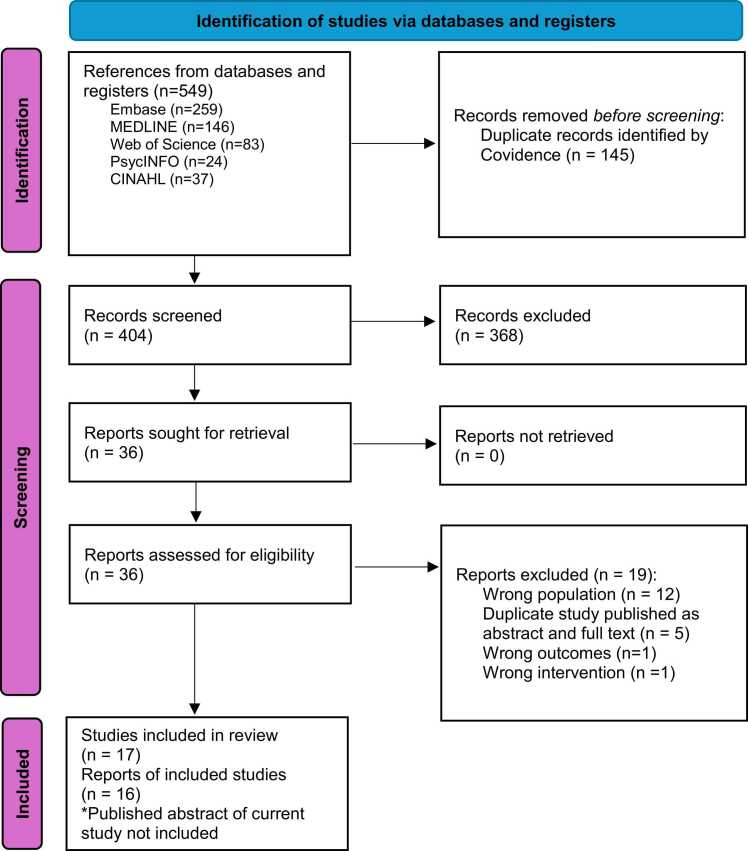
PRISMA Flow Diagram.

Zotero reference management software (Digital Scholar, Vienna, VA) was used to import and manage references. Results were exported into a systematic review manager, Covidence (Veritas Health Innovation). Two independent reviewers screened article titles and abstracts using the review’s inclusion criteria in Level 1 screening. Eligible sources were reviewed in full text in Level 2. Studies not meeting inclusion criteria were excluded with reasons for exclusion documented in the PRISMA Flow Diagram (Figure 1).^10^ Disagreements between two reviewers were resolved through discussions with the third reviewer.

### Data extraction and charting

Data extraction was completed by two independent reviewers. The following information was extracted in Covidence®: title, authors, publication year and location; study aim; sample characteristics; methodology and measures; and key findings as they related to the research questions. Any discrepancies between reviewers were resolved with the involvement of the third reviewer. An inductive content analysis approach was applied to organize the extracted data into themes, which are presented in the results section and Table 2. Initial coding was conducted by the first author and subsequently reviewed and verified by all members of the research team to ensure consistency and rigor.

**Table 2:** Literature Regarding Caregiver Experiences After Lung Transplantation

Author, Year, Location	Purpose	Sample	Design	Measures	Key Caregiver Experiences	Positive Caregiver Experience	Caregiver Challenges	Publication Type
Ågren et al., 2017^12^ Sweden	To describe anxiety, depression, stress, coping, and burden for patients and their NoK before and up to 2 years after LTx.	Adult patients (N=26 lungs) and their appointed NoK (N=18)	Quantitative, longitudinal study; survey	EuroQol 5-dimensional questionnaire; HADS; Impact of Event Scale; Mastery Scale; CBS	The score for the HADS anxiety subscale in the entire group of NoK was 7.4% at baseline and had decreased to 1.6% after 2 years. For the depression subscale, the score was 2.1% at baseline and 0.5% after 2 years. Anxiety/depression levels were higher when NoK had a lower education level (p=0.03).	NoK with a higher level of education had a higher coping ability (p=0.03).	22.9% of NoK scored medium or high levels of burden on the CBS, at baseline, decreasing to 9.1% after 2 years. General strain was most affected, with 22.3% recording medium or high levels of burden at baseline, decreasing to 6.9% after 2 years, followed by disappointment (17% at baseline, decreasing to 6.4% after 2 years).	Journal Article
Goetzmann et al., 2012^13^ Switzerland	To investigate the psychosocial and physical health of both patients and their spouses using a sample of heart-, lung-, liver-, and kidney-txpl patients.	Patients who had undergone a LTx (N = 42), as well as their spouses.	Quantitative, cross-sectional study	SF-36; Relationship Assessment Scale; Questions on Life Satisfaction Survey; Burnout Measure Scale	No difference between patients and caregivers for sense of coherence or life satisfaction Spouses felt significantly physically better than the control sample (norm value). Patients rated the quality of their relationship significantly higher than their spouses did.	Patients and their spouses possessed distinct personal resources (sense of coherence) and were more satisfied with life than the general population.		Journal Article
Haines, 2016^14^ USA	To assess stress and anxiety in post-LTx caregivers, and how these may be affected by re-hospitalization and/or development of complications. To gain an increased understanding of the post-Ltx experience from the perspective of the caregiver.	Convenience sample (N=60) of caregivers recruited from a tertiary medical center.	Mixed methods design	PSS, STAI, and interviews	Both the PSS and STAI scores were higher than normed group scores. Time since LTx and PSS scores were statistically significant (r=0.29; p=0.005) indicating the longer the caregiving experience, the greater the stress. Themes: The Honeymoon Phase. Themes: 1) Concern for recipient health 2) Concern for caregiver health and well-being 3) Not being prepared for the caregiving experience 4) Significant life changes made for the recipient			Conference Abstract
Rosenberger et al., 2012^15^ USA	To discuss psychosocial effects of txpl on patients' primary family caregivers, as caregivers play a major role in maintaining their loved ones' physical and mental health and also undergo significant exposure to the stressors of the txpl experience.	Caregivers of txpl recipients (N=178 recipients)	Literature review (narrative)	Not applicable	Exposure to the chronic stresses of txpl may cause caregivers of both lung candidates and recipients to experience a lower quality of life than non-caregiving adults.	Caregivers also reported positive outcomes of caregiving, such as discovering inner strength and support from others and realizing the important things in life.	Although performing caregiving activities can often be gratifying, adjusting to the changing family dynamics, household responsibilities, and job-related capacities associated with txpl may compound the discrete burdens of caregiving, as evidenced by caregivers reportedly spending less of their day in a positive mood than did the lung recipients for which they care.	Journal Article
Dew et al., 2010^16^ USA	To examine the effects of caregiver burden on caregivers of CTT recipients and whether any decrements in caregiver well-being affected patient outcomes.	302 consecutive CTT recipients, 269 (lung n=154) had a family caregiver who agreed to enroll	Quantitative study	Multiple QOL domains, caregiving burden and psychosocial factors at 2-, 7- and 12-months post-CTT.	Caregivers average emotional and social QOL exceeded normative levels at all time points. Physical functioning and bodily pain worsened over the year (p<0.01 and p<0.05, respectively).	Caregiver optimism at the initial assessment predicted QOL in all domains.	Greater caregiver burden predicted poorer physical and emotional QOL. Poorer family support predicted poorer emotional and social QOL (all p<0.001). Controlling for patients' physical functional status at 12-months post-CTT, caregivers' perceptions that their own health was poorer independently increased patient mortality risk during the next 8 years (p<0.05): for each 10-point drop on the 100-point health perceptions scale in caregivers, patient mortality risk increased by 20%.	Conference Abstract
Myaskovsky et al., 2012^17^ USA	To determine how the HRQOL of caregivers to CTT recipients changes over the first-year post-txpl and to compare the trajectory of change in HRQOL in caregivers of lung versus heart txpl recipients. To determine whether we can predict caregiver HRQOL at 1-year post-txpl with caregiver demographic and psychosocial factors assessed early after txpl. To determine whether caregiver HRQOL at 1-year post-txpl would predict subsequent CTT recipient survival.	Family caregivers of lung or heart txpl recipients (aged 18+), post txpl, beyond the first 2 months post-txpl (n=134).	Mixed methods study	Patient medical records; SF-36; Life Orientation Test; Sense of Mastery Scale; Brief COPE scale; Zarit Burden Interview	Caregivers of lung recipients indicated more txpl-related health worries than did caregivers of heart recipients. Although caregiver energy level (vitality) improved during the course of the year, their average level of physical functional impairment significantly worsened, as did their level of pain.	Among the demographic variables, younger caregivers reported better general health, physical functioning, and fewer role performance limitations due to physical problems. Among the psychosocial resources, greater optimism predicted greater vitality, and a stronger sense of mastery shortly after the txpl predicted greater general health and mental health. Among caregiver burden variables, less activities impairment predicted better physical functioning, and less perceived personal burden was associated with greater vitality and less bodily pain.		Journal Article
Xu et al., 2012^18^ USA	To identify the daily activities and burdens after LTx on recipients and their caregivers.	Dyads of LTx recipients and their family caregivers (n=21)	Qualitative	Semi-structured interviews	No activities were uniquely reported by LTx recipients or caregivers. Related activities were grouped into 13 main categories. Nearly one-third of reported activities (30%) were health-related and included medication taking, health monitoring, medical appointments, and therapy or exercise. The balance of activities was related to routine household and caregiving activities. During the interviews, participants often expressed more in-depth feelings and perspectives than the list of activities and emotions alone would indicate. Family caregivers reported a mixture of both positive and negative emotions.	Caregivers reported positive emotions more frequently than LTx recipients during their interviews, Both LTx recipients and caregivers spent less than 10% of their day in a bad mood. Caregivers associated positive emotions with the performance of activities more often than LTx recipients.	Caregivers reported less time spent per day in a very good mood (40% vs 60%). Specific caregiving tasks were typically rewarding for caregivers, but on the whole, care giving in combination with other roles and responsibilities such as household and job-related tasks, took its toll.	Journal Article
Haines et al., 2014^19^ USA	To test the feasibility of using MBSR techniques to decrease stress and anxiety in caregivers of LTx candidates/recipients who required admission to an acute care facility.	30 caregivers of patients admitted to an acute care LTx unit as a consequence of deterioration in their health status	Quantitative; pre-test/post-test design (watching DVD that demonstrated MBSR techniques) pilot study	Demographic data (age, gender, race, education, etc.), PSS, and STAI		PSS and STAI scores from pre- to post-intervention in those who watched some or none of MBSR DVD did not change significantly.	MBSR decreased, PSS scores and STAI scores decreased from pre- to post-intervention in those who watched the DVD.	Journal Article
Gerity et al., 2018^12^ USA	This quality improvement project evaluated whether a multimedia education method compared to standard education method improves post-txpl care knowledge, anxiety, and satisfaction with the education experience in LTx patients and their caregivers.	LTx patients and primary caregiver dyads (n=37 LTx patients, n=37 caregivers).	Quantitative, quality improvement project.	The study compared a historic control group who received the standard education method (n=19 dyads) and a multimedia group who received a multimedia education method (n=18 dyads). A satisfaction survey was completed by patients and caregivers at the end of the program.	A higher percentage of caregivers in the multimedia group in comparison to the standard group reported less anxiety about the surgery (65% vs 33%) and knowledge gains (94% vs 80%).	Caregivers and txpl team members indicated that the multimedia method was more effective than the standard method in improving post-txpl care knowledge and preparing caregivers for delivering care after the surgery. Most primary caregivers receiving the multimedia education method felt less anxiety about the txpl surgery, suggesting the method was also beneficial in reducing caregiver anxiety…A smaller percentage of caregivers (65%) in comparison to patients (71%) reported reduced anxiety after multimedia education.		Journal Article
Taylor, 2018^21^ Australia	To explore the expectations and needs of carers during the LTx journey and the types of intervention required by social work to determine if increased interventions would be of benefit in optimizing psychosocial wellbeing.	Caregivers of LTx recipient (n=73) were surveyed.	Quantitative, cross-sectional survey	16-item Likert scale questionnaire related to txpl assessment processes; information provided regarding the carer expectations and their relationships pre- and post-txpl; support by the multidisciplinary team or potential benefit from a carers’ support group.			85% of respondents felt that they would benefit from a carer-specific support group. 73% indicated that psychosocial assessment by the social worker would help address relationship issues. 68% of carers reported wanting more written information on their role and the expectations pre- and post-txpl.	Published Abstract
Li et al., 2016^22^ Australia	To determine if LTx patients and carers at a major LTx service have PC needs.	Patients (n=113) and carers (n=87) attending LTx clinics between April and October 2015.	Quantitative, prospective, cross-sectional study	QOL (Medical Outcomes Study Questionnaire SF-36), symptoms (Edmonton Symptom Assessment System), and supportive care needs (Carer Support Needs Assessment Tool)	LTx patients and carers do have substantial PC needs.	Incorporating PC into both pre- and post-LTx care may enhance symptom management and bolster psychosocial support for patients and carers.	Regardless of LTx status, a significant proportion of patients and carers indicated a need for more support, for example with prognostication (patients 42% and carers 51%) and coping (patients 28% and carers 37%).	Published Abstract
Yagelniski et al., 2020^23^ Canada	To explore experiences and perceptions of LTx caregivers identified from a satellite clinic to inform the development of educational resources.	Caregivers of LTx recipients (n=12).	Qualitative study with a phenomenology approach.	NVivo software was used to code data and identify themes.	Being a caregiver was stressful, and supports were necessary for those undertaking this role. Family and friend support and support from others in the same situation was particularly helpful. 4 emerging themes. (1) the stress of being a caregiver; (2) caregivers undertake a variety of roles; (3) caregivers require support; and (4) satisfaction with health care providers.	All caregivers expressed satisfaction with the level of care that they received from the surgical txpl center. Most participants felt adequately prepared for the procedure, were happy with their txpl education, and viewed the preoperative trip to the txpl center as extremely comprehensive and beneficial.	Caregivers identified several stressors during the txpl process and described various strategies for coping. Many caregivers also reported periods of anxiety throughout the entire process and indicated that stress, such as financial stress, does not end once the patient is transplanted.	Journal Article
Giordano et al., 2021^24^ USA	To explore family perceptions on how using txpl hospitality house accommodations impacted opportunities for family-centered care and affected physiological and physical security, as well as belonging and esteem.	71 lung candidates/recipients made up the majority of patient/caregiver groups (56%)	Qualitative study; focus groups and intensive interviews. A multi-stakeholder method.	Thematic Analysis	5 themes identified: 1. Perception of family-centered care 2. Perceptions of physiological needs 3. Perceptions of physical security 4. Perceptions of belonging 5. Perceptions of esteem	Patients and caregivers universally reported that they believed their health and recovery was enhanced by the experience of staying at a txpl hospitality house. The availability of the hospitality house to caregivers reduced stress and worry.		Journal Article
Glaze et al., 2021^25^ USA	To examine the experiences of caregivers of recipients pre-and post-LTx.	20 participants of caregivers and LTx recipients.	Qualitative exploratory study with semi-structured open-ended interviews.	Interviews were analyzed using conventional content analysis to allow codes and 4 themes to emerge.	4 main themes and 12 sub-themes were identified. The former included (1) establishing the diagnosis, (2) caregivers’ role, (3) caregivers’ psychological and psychosocial issues, and (4) support.	Support played an important role in caregiving experiences.	Caregivers lacked basic knowledge related to LTx. The caregivers' roles necessitated rearranging priorities, lifestyle changes, and redirecting emotional and physical energy.	Journal Article
Ivarsson et al., 2014^26^ Sweden	To describe relatives’ experiences before and during the patient’s hospital stay as well as during the first 6 months after a heart or LTx.	Relatives with a close relation to patients (n=15) who had undergone a heart or LTx about 6 months earlier. The relatives were chosen by the patients.	Qualitative content analysis (qualitative, descriptive and retrospective design)	Semi-structured interviews. The questions explored their experience of information and support during the waiting time until the present.	The relatives received support and practical assistance with the dependent children from other NoK and friends. The relatives spent significant time assisting the patient physically, psychologically or socially. The relatives described their social networks as being of great help in terms of social and practical assistance. Relatives described how they felt they got support from various activities, such as keeping pets, gardening, walking, choir and meeting other people. Most relatives felt support in some kind of faith or spirituality, in the form of a strategy to master the situation related to the txpl.	Relatives found it helpful to talk to someone with personal experience and felt inspired, hopeful and confident of the future. Only a few relatives had received assistance in getting this contact. Few relatives had met someone with personal experiences of txpl, but those who had felt it was a very positive opportunity.	Circumstances regarding dependent children: They described the importance of adequate information from health care professionals, given in an educational, age-appropriate and individual way without scaring the children. Some relatives described that they had not received any help from health services because the municipality’s assistance was limited only to the sick.	Journal Article
Song et al., 2010^27^ USA	To explore the meaning of chronic rejection after LTx from the perspectives of family and clinician caregivers and describe the impact of the onset and progression of chronic rejection on subsequent clinical management decisions and caregiving.	Family members (n=10) and clinician caregivers (n=3) of the txpl recipients	Qualitative; Grounded Theory	Semi- structured interviews with open-ended questions.	Meaning of chronic rejection described as inevitable, irreversible; unpredictable; and “going back to where we started". Until recipients were no longer competent, caregivers believed all treatment options (including re-txpl) had been exhausted, or suffering was prolonged, caregivers were reluctant to halt extraordinary treatment measures. Caregivers perceived that certainty regarding poor prognosis was required for palliative care and that palliative care was end-of-life care. Consequently, trials of aggressive treatment typically precluded palliative care. When families were asked how they made treatment decisions after the onset of chronic rejection, their responses were always couched in terms of respecting the recipient’s wishes to keep going, (such as re-txpl), and when recipients were no longer competent, to keep them comfortable.			Journal Article

CBS, Caregiver Burden Scale; CTT, cardiothoracic transplant; HADS, Hospital Anxiety and Depression Scale; HRQOL, health-related quality of life; LTx = lung transplantation; MBSR, mindfulness-based stress reduction; NoK, next of kin; PC, palliative care; PSS, Perceived Stress Scale; QOL, quality of life; SF-36, Short Form-36; STAI, State-Trait Anxiety Inventory; txpl = transplant.

## Results

### Sources of evidence inclusion

Studies identified and selected at each stage, and the number of articles excluded with reasons for exclusion, are presented in a PRISMA flow diagram in Figure 1. After removing duplicates from the initial 549 sources, 404 citations remained. Title and abstract screening (Level 1) excluded 368 citations, leaving 36 for full-text review. Level 2 screening excluded 19 sources with 14 due to non-relevant population, outcomes, or intervention. An additional five sources were identified as “overlapping” or duplicate where the same study was published in different formats (e.g., conference abstracts and full articles) or where several publications used the same dataset. To avoid duplication and ensure accuracy, only one full article was included in such cases. Seventeen sources met the inclusion criteria. The published conference proceeding abstract of this scoping review was excluded to avoid duplication, resulting in a final inclusion of 16 sources.

### Characteristics of included sources

**Figure 2:**
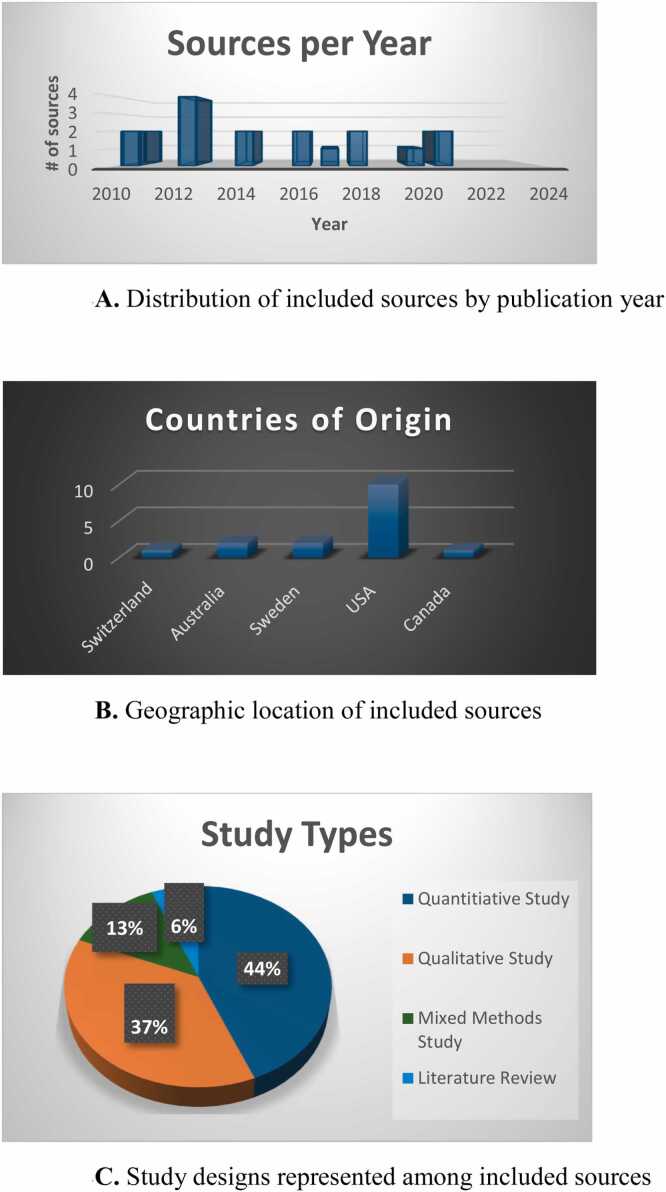
Summary Statistics of Characteristics of 16 Sources of Evidence Included in the Scoping Review. B. Geographic location of included sources. A. Distribution of included sources by publication year. C. Study designs represented among included sources.

Summary statistics of the 16 included sources are depicted in Figure 2: 75% were classified as publications (n = 12), 19% peer-reviewed conference abstracts (n = 3), and 1 poster presentation. Most sources were based in North America (11/16 = 69%), followed by Europe or Australia. Only 19% (3/16) were published within the past 5 years. Seven sources were qualitative studies (44%), 6 quantitative (37%), 2 mixed methods (13%), and 1 narrative literature review. Sample size of informal caregivers ranged from small (≤ 15) to large (≥ 100) across publications.

### Review findings

**Figure 3:**
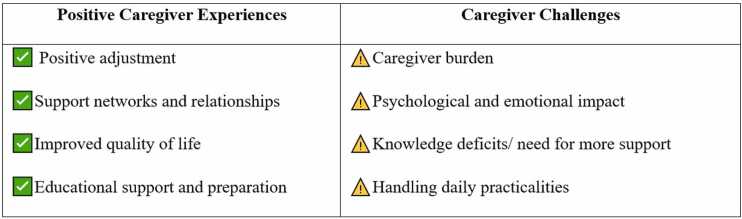
Informal Caregiver Experiences Identified After Lung Transplantation.

The purpose of 16 included sources focused on (1) investigating the psychosocial health and well-being of caregivers^12^, ^13^, ^14^, ^15^; (2) caregiver burden and quality of life^12^, ^16^, ^17^, ^18^; (3) interventions, education and support for caregivers^19^, ^20^, ^21^, ^22^, ^23^, ^24^; and (4) overall family and caregiver experiences in the transplant process.^25^, ^26^, ^27^ The themes encompassed positive and challenging experiences, as depicted in Figure 3.

### Positive caregiver experiences

Positive caregiver experiences were identified in 11 sources^12^, ^13^, ^16^, ^17^, ^18^, ^19^, ^20^, ^23^, ^24^, ^25^, ^26^ including 4 main themes: positive emotional adjustments; support networks and relationships; improved health outcomes; educational support and preparation.

#### Positive emotional adjustments

Caregivers of LTx recipients reported positive emotional adjustments including personal growth, resilience, and satisfaction.^13^, ^16^, ^17^, ^18^, ^20^, ^23^, ^26^ Caregivers experienced satisfaction from supporting loved ones and experienced positive emotions, during daily caregiving responsibilities including health-related tasks, household chores, and errands,^18^ with less than 10% of their day feeling in a low mood.^18^ Optimism predicted better physical and mental health-related quality of life,^16^ higher vitality, and greater sense of control.^17^ Educational interventions were associated with reduced anxiety and improved emotional well-being.^20^ Caregivers demonstrated coping abilities and reported higher life satisfaction compared to the general population.^13^, ^17^ Many experienced personal growth, resilience, and caregiver identity through new skills, competencies, and complex medical care responsibilities.^26^

#### Support networks and relationships

Support networks and relationships were described in 6 studies as helping participants navigate their caregiving journey.^20^, ^23^, ^24^, ^25^, ^26^ Having extended support networks of loved ones nearby benefited caregivers' emotional well-being.^24^, ^26^ Hospitality houses for patients and caregivers improved access to housing, food, and reduced stress,^24^ while improving overall well-being, and developing a sense of community and belonging.^24^ Other activities included pet care, gardening, walking, singing in a choir, and socializing.^26^ Speaking with someone with personal transplant experience helped caregivers feel inspired, hopeful, and confident about the future.^24^, ^26^ Strong caregiver-provider communication^23^ helped caregivers feel well-prepared for transplant.

#### Improved caregiver health outcomes and quality of life

Improved caregiver health outcomes and quality of life refer to positive changes in participants' overall health and wellbeing were described in 6 studies.^12^, ^13^, ^16^, ^17^, ^22^, ^24^ In a Swiss sample, caregiving spouses scored higher on the physical component scale of the Quality of Life questionnaire compared to norm values from European populations.^13^ Caregivers' emotional and social quality of life was above normative levels though their physical functioning and bodily pain declined over the year after transplant.^13^ General health, physical functioning, and role performance of LTx caregivers improved, especially for younger caregivers who had better overall health, physical functioning, and fewer role limitations in the US sample of family caregivers.^17^ Palliative care was important after transplant, as it contributed to better symptom management and increased psychosocial support for both patients and caregivers.^22^ Caregivers in hospitality housing also reported reduced financial stress and enhanced community support.^24^

#### Educational support and preparation

Educational support and preparation were recognized in 3 sources.^20^, ^23^, ^26^ Caregivers and transplant team members noted that multimedia approaches, delivered by coordinators using visual presentations, instructional videos, and hands-on demonstrations, were more effective than traditional methods in enhancing post-transplant care knowledge and post-surgery care.^20^ Caregivers receiving multimedia methods were satisfied with their transplant experience and experienced less anxiety.^20^ Relatives who connected with someone with personal transplant experience found it inspiring, hopeful, and reassuring.^26^ Participants felt well-prepared, and were satisfied with their transplant education, and preoperative visits to the transplant center.^23^

### Challenges of caregiving after LTx

Informal caregivers described challenging experiences in 14 out of 16 sources^12^, ^14^, ^15^, ^16^, ^17^, ^18^, ^19^, ^21^, ^22^, ^23^, ^24^, ^25^, ^26^, ^27^ including caregiver burden, stress, knowledge deficits and the need for more education and support in handling multiple daily practicalities.

#### Caregiver burden

Caregiver burden was described in 5 sources as the experiences of physical, emotional, and general strain related to caregiving, leading to long-term emotional and physical challenges.^12^, ^15^, ^16^, ^17^, ^25^ For example, 22.9% of next-of-kin caregivers scored medium or high levels of burden on the Caregiver Burden Scale at baseline, decreasing to 9.1% after 2 years.^12^ Greater caregiver burden was linked to poorer quality of life for both caregivers and patients.^16^ Caregivers' belief that their own health was poor independently increased the risk of patient mortality over the following 8 years.^16^ Caregivers described feeling overwhelmed by the complexities and demands, particularly during early post-transplant periods.^25^

#### The psychological strain of caregiving

Psychological strains of caregiving refer to the negative effects such as emotional distress, anxiety and stress that caregiving had on psychological and emotional wellbeing was evidenced in 8 sources.^12^, ^14^, ^15^, ^16^, ^17^, ^18^, ^23^, ^25^ The transplant process worsened caregivers' stress levels. The Perceived Stress Scale and State-Trait Anxiety Inventory scores were higher in LTx caregivers compared to normed groups.^14^ Caregivers reported spending less time each day in a very good mood compared to LTx recipients (40% vs 60%).^18^ Caregivers of LTx recipients expressed more transplant-related health concerns than those caring for heart transplant recipients.^17^ Caregivers experienced anxiety including financial stress, which continues post-transplant.^23^ Caregivers described feeling emotionally drained with symptoms of depression and feeling isolated from their usual support systems.^25^

#### Knowledge deficits and the need for more support

Knowledge deficits and more support refer to caregiver’s insufficient or absent knowledge to effectively carry out caregiving tasks and need for more healthcare system support. In 7 sources, caregivers expressed needing additional support, including prognostication, coping, and guidance throughout the transplant journey.^21^, ^22^, ^23^, ^25^, ^26^, ^27^ Patients and caregivers expressed needing additional support, particularly with prognostication (42% patients, 51% caregivers) and coping (28% patients, 37% caregivers).^22^ Caregivers (85%) believed they would benefit from support groups, and 73% felt that psychosocial assessments by social workers could help address relationship challenges.^21^ Caregivers reported feeling unprepared about LTx and their caregiving responsibilities,^25^ and desired written information regarding their role and expectations before and after transplant.^21^ Swiss caregivers noted they had not received practical assistance (e.g., help caring for dependent children) from healthcare services, as municipal supports were limited to the sick.^26^ Caregivers expressed confusion about complex medication regimens and uncertainty when contacting healthcare providers.^23^, ^27^

#### Handling daily practicalities

Handling daily practicalities refers to LTx caregivers’ challenges managing time to complete caregiving roles alongside multiple roles and responsibilities in their personal and professional lives - as described in 6 sources.^18^, ^23^, ^25^, ^26^, ^27^ Caregivers struggled to balance caregiving with other responsibilities, including household and job-related tasks.^18^ Caregivers dedicated significant time assisting patients in physical, psychological, and social ways.^26^ Caregivers faced challenges managing family dynamics, involving chronic rejection or end-of-life decisions. Families were asked about treatment decisions following chronic rejection, and responses emphasized the recipient’s wishes to continue, opting for re-transplantation, and comfort care when the recipient was no longer capable.^27^ One study outlined 13 specific activities related to the challenges of handling daily practicalities including coordinating multiple appointments, complex medication schedules, transportation, and household responsibilities while caring for the transplant recipient.^18^ Caregivers reported disruptions to their work schedules and careers, requiring extended leaves or early retirement.^25^

## Discussion

This scoping review represents a comprehensive examination of the literature surrounding informal caregiving experiences after LTx. Despite this systematic search spanning 15 years, only 16 sources met the inclusion criteria, highlighting limited evidence in this aspect of transplant care, and the urgent need for caregiver-focused research.^28^ Informal caregivers play crucial roles in LTx, however, few interventions address these specific challenges, with efforts limited to educational approaches or mindfulness-based stress reduction.^29^ Informal caregivers of LTx recipients experience positive and challenging experiences. Positive experiences—emotional resilience, supportive relationships, educational preparation, and personal growth—suggest potential “benefit finding” in the caregiver's role. “Benefit finding” refers to experiencing personal growth or positive adaptations responding to major stress or trauma^28^ including higher life satisfaction among caregivers; however, its role among LTx caregivers remains uncertain.^28^ Positive caregiver experiences may be enhanced through targeted interventions including multimedia educational programs, hospitality housing, and peer support networks.

Conversely, challenges identified - caregiver burden, psychological strain, knowledge deficits, and difficulties managing daily practicalities—are consistent with other LTx literature focusing on candidates and recipients.^29^, ^30^, ^31^ Fifteen percent of LTx candidates' caregivers reported their physical health worsened since caring for the patient and 22% reported fatigue, worsening health, and poorer quality of life.^31^ A recent literature review on the caregiver burden described that LTx caregivers experience significant psychological, physical, and financial burdens.^30^ Poor health amongst caregivers has been associated with an increased risk of poor outcomes and even mortality in LTx recipients.^16^, ^30^

Nineteen percent of sources were published in the past 5 years, highlighting the need for new research. Changes in transplantation practices and patient demographics emphasize the need to comprehensively redefine transplant benefits, including patient- and caregiver-centered outcomes beyond traditional survival metrics.^32^

The predominance of North American studies (69%) suggests potential cultural and health care system biases. Informal caregiving experiences may differ across cultural, systemic, and socioeconomic contexts. Socioeconomic status should not exclude patients from transplant candidacy, urging centers to consider broader support networks and psychological resources.^5^

The methodological diversity of included studies offers valuable insights; but complicates synthesis and comparison. Studies were small, single-center observational designs, limiting generalizability of findings and precluding meta-analysis. Low transplant volumes and varying standards of care hinder large, prospective multicenter studies in transplant research. Limited studies showed promise for multimedia educational approaches and mindfulness-based interventions,^19^, ^20^, ^28^ but significant gaps exist in caregiver intervention research.^28^

Caregiver support impacts quality-of-life and clinical outcomes after LTx. Patients with non-spousal primary caregivers, such as adult children, have poorer overall survival,^33^ highlighting the need for tailored support across caregiver types.

## Implications for research

This scoping review highlights areas for future research to better understand and support informal caregiving after LTx. Longitudinal and multicenter studies are needed to assess caregiver burden and its impact on caregiver and patient outcomes. Given that most caregiver burden assessment tools have been adapted from non-LTx populations, rigorous psychometric validation tailored to the unique experiences faced by LTx caregivers is imperative to ensure accurate and meaningful assessment.^30^ Mixed methods and dyadic approaches are warranted to capture the complex, reciprocal dynamics between patients and caregivers. Given the scarcity of intervention studies, priority should be placed on developing and testing multicomponent interventions ranging from educational and psychosocial supports to peer and practical assistance. Future caregiver research can explore telehealth and technology-driven solutions, including mobile applications and virtual reality training.^34^ Including patient and caregiver inputs is essential to shape patient-related outcome measures^32^ and caregiver-specific metrics reflecting caregivers’ experiences.^29^

## Limitations

To the authors’ knowledge, this is the first scoping review exploring informal caregiving after LTx. Limitations include restricting sources to the past 15 years, which may overlook historical shifts in transplantation and caregiving. Limiting to English-language sources risks selection bias, as caregiver research is culturally and geographically diverse. Most available studies were observational, single-center, early-phase, and lacked replication. Cross-sectional designs precluded incidence estimates and causal inference. Several sources were conference abstracts with minimal methodological detail. Finally, the absence of methodological appraisal and risk-of-bias assessment reflects inherent limitations of scoping review methodology.^35^

## Conclusion

Our findings reveal positive and challenging caregiver experiences. Informal caregivers demonstrate resilience and derive meaning from their roles, while facing significant physical, psychological, and practical challenges. While informal caregivers experience substantial challenges, targeted interventions may support their well-being and ability to provide care. This scoping review serves as a call to action for the LTx community to prioritize caregiver research. We must ensure that informal caregivers receive the support they need to maintain their own wellbeing while providing optimal care for LTx recipients.

## Funding sources

This work did not receive any specific grant from funding agencies in the public, commercial, or not-for-profit sectors.

## CRediT authorship contribution statement

Each author contributed substantially to the conception and drafting of the manuscript and qualifies for authorship. All authors take responsibility for the contents of this manuscript. JS, JC, EC, and JR contributed to the conceptualization of the scoping review; JS, JC, EC, JR, and DS contributed to the data extraction and analysis; and JS, JC, EC, JR, and KB contributed to the writing of the manuscript. All authors gave approval for the publication.

## Declaration of generative AI and AI-assisted technologies in the writing process

During the preparation of this work, the author(s) used ChatGPT to improve the language and readability. After using this tool/service, the author(s) reviewed and edited the content as needed and take(s) full responsibility for the content of the publication.

## Declaration of Competing Interest

The authors declare that they have no known competing financial interests or personal relationships that could have appeared to influence the work reported in this paper.
